# Conformational sampling of CpxA: Connecting HAMP motions to the histidine kinase function

**DOI:** 10.1371/journal.pone.0207899

**Published:** 2018-11-29

**Authors:** Nathalie Duclert-Savatier, Guillaume Bouvier, Michael Nilges, Thérèse E. Malliavin

**Affiliations:** 1 Unité de Bioinformatique Structurale, Institut Pasteur and CNRS UMR3528, Paris, France; 2 Centre de Bioinformatique, Biostatistique et Biologie Intégrative, Institut Pasteur and CNRS USR3756, Paris, France; Danish Cancer Society Research Center, DENMARK

## Abstract

In the histidine kinase family, the HAMP and DHp domains are considered to play an important role into the transmission of signal arising from environmental conditions to the auto-phosphorylation site and to the binding site of response regulator. Several conformational motions inside HAMP have been proposed to transmit this signal: (i) the gearbox model, (ii) *α* helices rotations, pistons and scissoring, (iii) transition between ordered and disordered states. In the present work, we explore by temperature-accelerated molecular dynamics (TAMD), an enhanced sampling technique, the conformational space of the cytoplasmic region of histidine kinase CpxA. Several HAMP motions, corresponding to *α* helices rotations, pistoning and scissoring have been detected and correlated to the segmental motions of HAMP and DHp domains of CpxA.

## Introduction

The two-component signaling systems (TCS) are ubiquitously used by prokaryotes to sense and respond to various changes in environmental conditions [1]. TCS control a large diversity of cellular functions in bacteria [2–8] and can also regulate virulence and pathogenicity [9–13].

In general, a two-component system is composed of two proteins, a sensor histidine kinase (HK) and a response regulator (RR) [14]. Histidine kinases are multifunctional enzymes that share a conserved intracellular catalytic core linked to highly diverse signal sensors. By adopting different conformational states, histidine kinases are able to catalyze three different reactions: (i) upon signal sensing they activate an autokinase activity, eventually phosphorylating a conserved histidine residue, (ii) in the phosphotransferase state, the phosphoryl group is then transferred to a residue in the cognate response regulator to initiate the bacterial response, (iii) along an overall reverse direction, most histidine kinases are also able to dephosphorylate the response regulator once the pathway is shut down. The large majority of histidine kinases, the so-called class I HKs [15], are homo-dimeric membrane proteins in which the cytoplasmic region contains two distinct functional domains: an N-terminal dimerization and histidine phospho-transfer (DHp) domain and a C-terminal catalytic and ATP-binding (CA) domain. The DHp domains, which bear the Histidine phosphorylated in the presence of ATP, form a stable dimer [16]. In many histidine kinases, the DHp domain is preceded by a linker HAMP domain consisting of a parallel four *α*-helix bundle that operates as a conformational signal converter [17–19]. The name HAMP is an acronym formed using the first letter of proteins containing this domain: Histidine kinases, Adenylate cyclases, Methyl accepting proteins and Phosphatases. In several histidine kinases, a domain HAMP is the closest to the membrane, and receives the conformational perturbation arising from the extracellular sensor domain through transmembrane *α* helices.

The precise description at atomic level of the HAMP conformational motions involved in signal transmission is still debated. Several structures of histidine kinases determined so far [2, 20–28], provide significant support to a mechanistic hypothesis according to which interconversion between different functional states in histidine kinases is mediated through helical rearrangements (*α* helix rotation, scissoring, pistoning and bending) within the dimer of HAMP domains [29]. On the other hand, the dynamic bundle model in which one signaling state of HAMP is more disordered than the other has been also proposed [30, 31]. The study of an isolated HAMP dimer with systematic modifications of the protein primary sequence permitted [17, 32–34] to propose the gearbox model, in which two distinct conformations: the complementary x-da and the knobs-into-holes [17], correspond to different activities of the histidine kinase. Complementary x-da and knobs-into-holes states differ by various rotations of the *α* helices around the central axis of the *α* helices bundle, and are characterized by different profiles of the corresponding Crick angle deviation [34]. In agreement with the hypothesis of several conformations populated by HAMP, an EPR study of HAMP domain [35] gave dynamical evidences for a conformational equilibrium between two conformational states of HAMP.

Several bioinformatics studies intended to propose models for the internal communication within the proteins and the protein domains inside two component systems [36–38] and to describe the various conformational states of these systems [39, 40]. The metadynamics approach has been recently used [41] to investigate the conformational space of an isolated HAMP domain, exploring several rotation, piston and scissoring motions of the *α* helices.

X-ray crystallographic studies conducted on a three-unit poly-HAMP chain [18] and on chimera proteins with artificially introduced several HAMP domains [19] revealed that successive HAMP domains adopt several conformations, characterized by different packing arrangements as well as by variation of the crossing angles of *α* helices. These variations of conformations were proposed to underly the transmission of conformational variations along the histidine kinase. A more recent X-ray crystallographic structure of the *E coli* nitrite/nitrate sensor histidine kinase NarQ [42] diplays scissor motions of the average helix axes of C terminal extremities of HAMP, when the ligand binds to the sensor domain.

**Fig 1:**
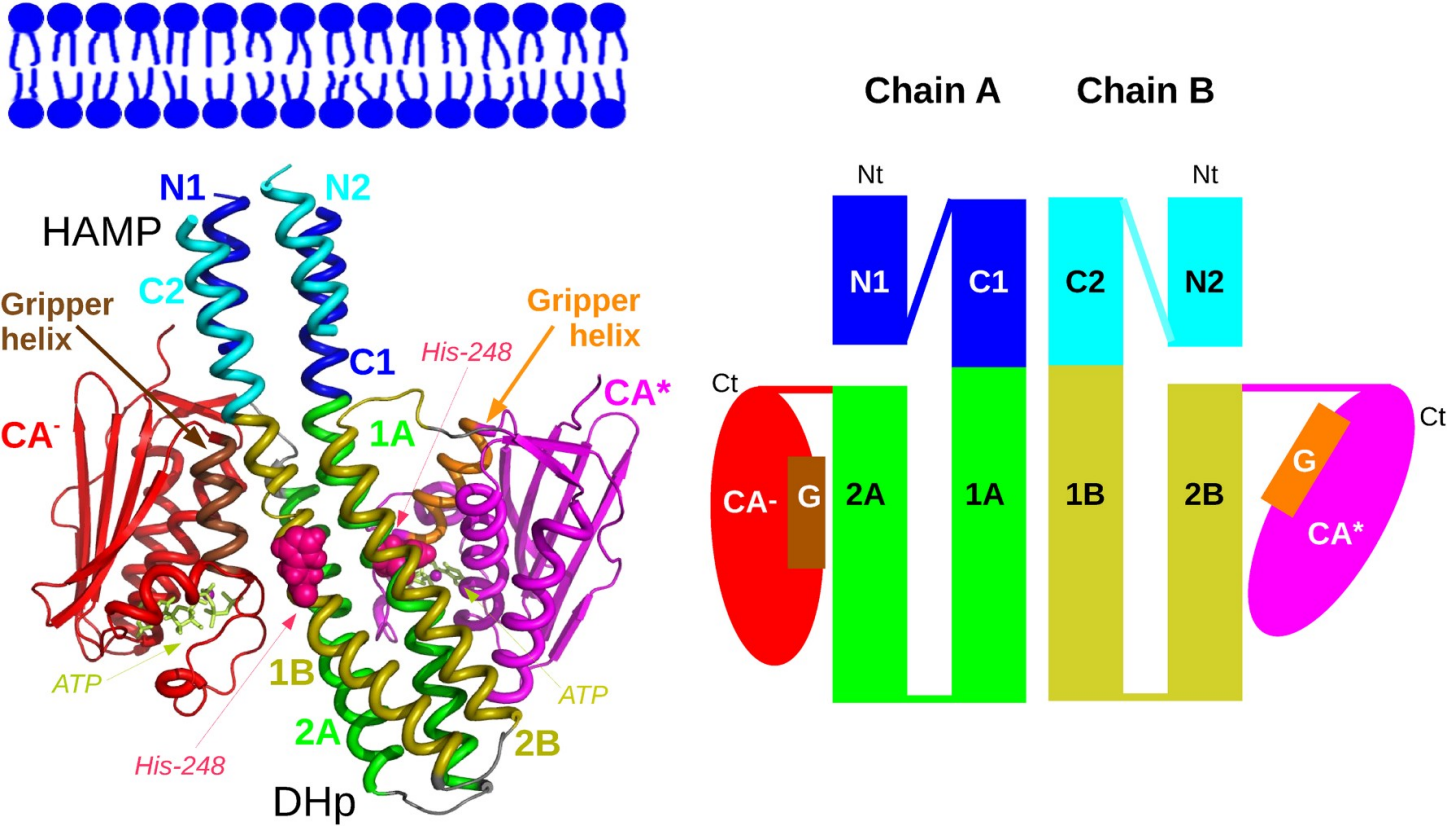
X-ray crystallographic structure of CpxA. Left panel. X-ray structure 4BIV [26] of CpxA shown in cartoon using PyMOL [62]. The approximate position of the membrane is indicated at the top of the figure. The loops of residues 202-218 connecting the HAMP helices are not shown for a sake of clarity. The *α* helices of HAMP and DHp and the CA domains used for defining the collective variables, are differently colored, in blue (chain A) and cyan (chain B) in HAMP and in green (chain A) and yellow (chain B) in DHp. The domain CA^−^ (red) (chain A) is moved apart from H248 in order to allow the transfer of phosphate group to the response regulator, whereas the domain CA* (magenta) (chain B) is in autokinase configuration, close to H248. The H248 are drawn in CPK and colored in purple, the ATP molecules are drawn in sticks and colored in lime. The gripper helices (residues 420-430 in domains CA* and CA^−^) are colored respectively in orange and brown. Right panel. Schematic representation of CpxA dimer: the HAMP and DHp *α* helices and the domains CA are represented with the same names and color-code than in the left panel. The gripper helices are labeled with the letter G. The N terminal and C terminal extremities are indicated with labels Nt and Ct.

The present study is devoted to the CpxA histidine kinase. The CpxA signaling system from *E Coli* mediates environment stress response which is essential for virulence and antibiotic resistance [43]. In the X-ray crystallographic structure of CpxA histidine kinase (Fig 1), the central domains DHp and HAMP display an asymmetric arrangement: segmental motions of these two domains were proposed [26, 44] to be related to the transition between different states of the CA domains (S1 Fig). In the following, the CA domains will be denoted as: the CA* domain positioned close to H248 for the autokinase reaction and the CA^−^ domain positioned apart from H248 [44], in order to allow interaction of H248 with the response regulator. We intend to explore the conformational space of cytoplasmic domain of CpxA using temperature-accelerated molecular dynamics (TAMD) [45], an enhanced sampling approach. We will use as collective variables geometric centers of HAMP and DHp *α* helices as well as geometric centers of the CA* and CA^−^ domains. The geometric centers of HAMP *α* helices have been chosen to explore the range of possibilities for the relative motions of the helices, without focusing on specific relative motions as this was the case in the Ref. [41]. The choice of geometric centers of *α* helices in DHp is justified by the intention to sample various orientations of DHp and HAMP without directing the system through a specific path. Our purpose is to examine which kinds of motions the HAMP *α* helices sample and how these motions are qualitatively correlated to motions of other protein domains more directly related to the various states of histidine kinase.

The enhanced sampling approaches [46, 47] have known a large development during the last decades, and are nowadays used for exploring many aspects of the functional dynamics as biomolecular conformational transitions [48], or protein-ligand interactions [49]. Metadynamics [50], accelerated molecular dynamics [51], generalized-ensemble molecular dynamics [52] are among the most frequently encountered enhanced sampling approaches. Other approaches, the temperature-accelerated molecular dynamics (TAMD) [53] and d-AFED [54] are based on the introduction of a supplementary equation calculating the evolution of the target values of the collective variables. This equation is coupled to a Langevin thermostat at an artificial temperature higher than the temperature of the MD simulation thermostat. This larger artificial temperature induces conformational transitions of the system, while the large artificial friction associated to the thermostat guarantees that the transition occurs adiabatically. The TAMD approach is considered [55] as part of methods that achieve enhanced sampling by using mean forces. TAMD has been used in the last years on various biological systems [48, 56–61] where it demonstrated its efficiency in accelerating the exploration of the conformational space of biomolecules.

## Results

### Variability of the global architecture of CpxA

**Fig 2:**
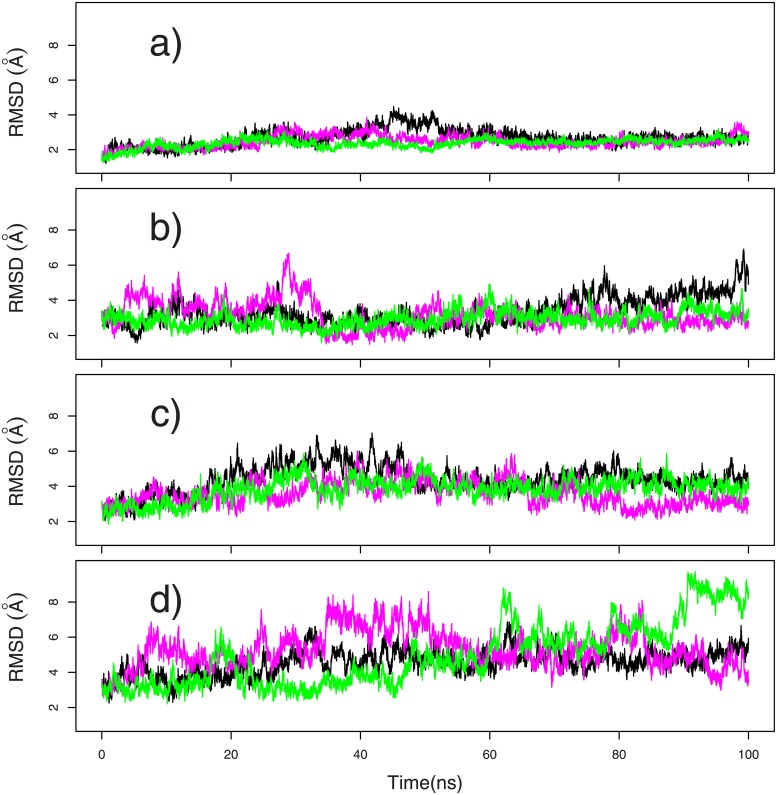
Coordinate root-mean-square deviations (RMSD, Å) on the CpxA dimer. The coordinate RMSD are calculated on heavy atoms of the protein backbone with respect to the conformation of the PDB entry 4BIV. The deviations are calculated for the following three-times repeated trajectories: (a) MD, (b) dbl15, (c) mid15, (d) tet20. On each plots, the different curves correspond to different replicas and the same color/replica correspondance will be used in the following.

The system mobility was monitored by calculating the coordinate RMSD (Å) with respect to the conformation of the PDB entry 4BIV (Fig 2). The three MD trajectories (Fig 2a) display a uniform RMSD at about 3 Å, proving the system stability. Along TAMD trajectories (Fig 2b–2d), the RMSD increases with the artificial temperature, in agreeement with the larger amount of energy introduced into the system.

For the TAMD trajectories dbl15 and mid15 (Fig 2b and 2c), defined in Methods, the global RMSD is stabilized at around 4-5 Å, whereas for the trajectory tet20 (Fig 2d), the RMSD value attains a plateau around 6 Å, and transiently jumps over 8 Å. The overall mobility of the CPxA dimer was further investigated by calculating average RMSD values on the individual domains of the histidine kinase: the HAMP dimer, the DHp dimer and the catalytic domains CA^−^ and CA* (Table 1). All RMSD values are in the range 0.8-2.1 Å, except the HAMP RMSD along dbl15 and tet20. Along MD, the domains HAMP and DHp can be considered as almost rigid, with individual RMSD values in the 0.8-1.0 Å range, whereas the domains CA^−^ and CA* displays larger RMSD respectively in the range 1.0-2.1 and 1.6-1.9 Å. Along the trajectories tet20, the increase of artificial temperatures induces a large increase of the HAMP RMSD, which reaches 4.0 Å. Nevertheless, the RMSD for HAMP along dbl15, stays in the range 1.3-2.8 Å, and is even smaller for mid15. Along TAMD trajectories, the DHp, CA^−^ and CA* domains RMSD vary similarly as in the MD trajectories. Overall, along the same trajectory, individual RMSD are smaller than the global RMSD (Fig 2). This is the sign that the overall large mobility of the full cytoplasmic domain arises mainly from the variability of the relative positions of the domains of CpxA.

**Table 1:** Atomic coordinate RMSD (Å) averaged along trajectories for different CpxA regions. The RMSD were calculated on heavy atoms of the protein backbone by superimposing each analyzed domain on the corresponding one in the conformation of CpxA in the PDB entry 4BIV.

Region	MD	dbl15	mid15	tet20
HAMP	0.8 ± 0.1	2.3 ± 0.9	1.7 ± 0.4	3.2 ± 0.8
	0.8 ± 0.1	1.3 ± 0.5	1.5 ± 0.3	4.0 ± 1.1
	0.8 ± 0.1	2.8 ± 0.5	1.4 ± 0.4	2.9 ± 0.9
DHp	0.9 ± 0.1	1.0 ± 0.2	1.0 ± 0.2	1.1 ± 0.2
	1.0 ± 0.1	0.9 ± 0.1	1.0 ± 0.2	1.1 ± 0.2
	1.0 ± 0.1	1.5 ± 0.3	0.9 ± 0.1	1.1 ± 0.2
CA^−^	1.0 ± 0.1	1.0 ± 0.1	1.2 ± 0.2	1.3 ± 0.2
	1.9 ± 0.2	1.3 ± 0.3	1.1 ± 0.1	1.1 ± 0.1
	2.1 ± 0.4	1.1 ± 0.1	1.1 ± 0.1	1.2 ± 0.2
CA*	1.9 ± 0.4	1.4 ± 0.1	2.1 ± 0.6	1.6 ± 0.2
	1.6 ± 0.2	1.5 ± 0.2	1.3 ± 0.1	1.2 ± 0.1
	1.6 ± 0.2	1.3 ± 0.2	1.3 ± 0.1	1.4 ± 0.4

The reliability of HAMP dimer conformations was investigated by calculating the two following parameters (Table 2). First, the average distance between the geometric centers of the HAMP *α* helices of each monomer gives an indication on the overall stability of the dimer. Second, the percentage of *α* helix for HAMP residues 189-203 and 217-231 in each dimer chain gives information on the propensity of *α* helices to unfold. Unsurprisingly, the inter-monomer distance increases along TAMD trajectories with the artificial temperature. Along mid15 trajectories, the use of a restraint located at the middle of HAMP *α* helices, slightly decreases the inter-monomer distance with respect to dbl15. The largest observed average value for the inter-monomer distance is 10.3 Å in the second replica of tet20. The values for inter-monomer distance obtained from the trajectories were compared to the equivalent distances measured on polyHAMP chains, in PDB structures 3lnr [18] and 4i3m, 4i44 [19], and on the histidine kinase NarQ, in PDB structures 5jef, 5jeq [42]. In these structures, distances in the range of 8.4-10.3 Å have been measured, the largest distances being observed in the HAMP1 domain of 4i3m and 4i44 [19]. Thus, the upper limit of 10.3 Å corresponds to a possible conformation of HAMP domain already observed in X-ray structures and the observation of such average value along the trajectories validate the association level of HAMP dimer in the present work.

**Table 2:** Parameters describing the HAMP architecture calculated along each trajectory replica. The inter-monomer distance (Å) is calculated as the distance between geometric centers of C*α* from residues 189-203 and 217-231 of HAMP *α* helices in each monomer. The percentage of *α* helix is calculated on the same residues.

Parameter	MD	dbl15	mi15	tet20
HAMP Inter-monomer distance (Å)	8.4 ± 0.2	9.1 ± 0.5	8.8 ± 0.4	9.8 ± 0.6
	8.5 ± 0.2	8.7 ± 0.3	8.6 ± 0.3	10.3 ± 0.9
	8.4 ± 0.2	9.5 ± 0.5	8.8 ± 0.3	9.8 ± 0.7
*α* helix percentage in HAMP helices	86.7	87.0	83.1	75.9
	87.0	89.8	81.0	74.9
	87.1	87.4	84.7	79.5

The percentages of *α* secondary structure in the HAMP domain display values all larger than 80% except for tet20, where they are around 75% (Table 2). The global secondary structures are thus conserved in the HAMP dimer. Along the mid15 trajectories, the use of restraints connecting residues located at the middle of HAMP *α* helices has the effect to slightly reduce the percentage of *α* helix with respect to dbl15. Generally, along all MD and TAMD trajectories, the percentage of *α* secondary structure in HAMP dimer conformation agrees with the knowledge gathered from X-ray crystallographic structures and thus represents a good modeling of the HAMP dimer variability.

The variation of the CPxA global architecture has been monitored (S1 Fig) by comparing the two angles *θ*_*HAD*_ and *θ*_HA*D_ defined by the three geometric centers of HAMP-CA^−^-DHp and HAMP-CA*-DHp. In the X-ray crystallographic structure 4BIV, these angle values were respectively 116.6° and 105.5°. Along all trajectories, the angle *θ*_*HAD*_ is mostly larger than *θ*_HA*D_ (S2 Fig), but the two angles display a slightly more symmetric distribution in TAMD trajectories dbl15 (S2b Fig) and tet20 (S2d Fig) than in MD (S2a Fig) and mid15 (S2c Fig) trajectories. The symmetry of the distribution is obviously correlated to the ratio *θ*_*HAD*_/*θ*_HA*D_ between the two angles HAMP-CA^−^-DHp and HAMP-CA*-DHp (S3 Fig). Conformations corresponding to ratio values close to the minimum or maximum observed value have been extracted from each trajectory replica (S4 Fig). Along TAMD trajectories dbl15 and tet20, conformations with maximum ratio display a break between the axes of HAMP and DHp domains. Large ratios are the sign of asymmetry in HAMP and DHp arrangement in the dimer, reminiscent of the segmental motion proposed by Mechaly et al [26] (S1 Fig). At the contrary, small ratios correspond to more symmetric HAMP/DHp architecture. The trajectories MD and mid15 display less conformational variations between maximum and minimum ratio values, in agreement with the corresponding distributions of *θ*_*HAD*_/*θ*_HA*D_ (S2 Fig).

The internal mobility of the CpxA dimer is driven by the variation of relative positions of the domains HAMP, DHp, CA^−^ and CA*. HAMP displays the largest RMSD along trajectories, but nevertheless retains the features of a conformation in agreement with the observations made on X-ray crystallographic structures. Indeed, the inter-monomer distance within HAMP as well as the percentage of *α* helix monitored along the TAMD trajectories, agree with a stable HAMP dimer in agreement with X-ray crystallographic structures of CpxA. This validates the use of TAMD trajectories to explore the CpxA conformation space. Along trajectories dbl15 and tet20, large variations of the ratio *θ*_*HAD*_/*θ*_HA*D_leads to the appearence of conformations displaying segmental motions of HAMP and DHp domains, corresponding to asymmetric and more symmetric HAMP/DHp orientations, in agreement with the observations of Mechaly et al [26, 44].

### Relative motions of *α* helices within HAMP

**Fig 3:**
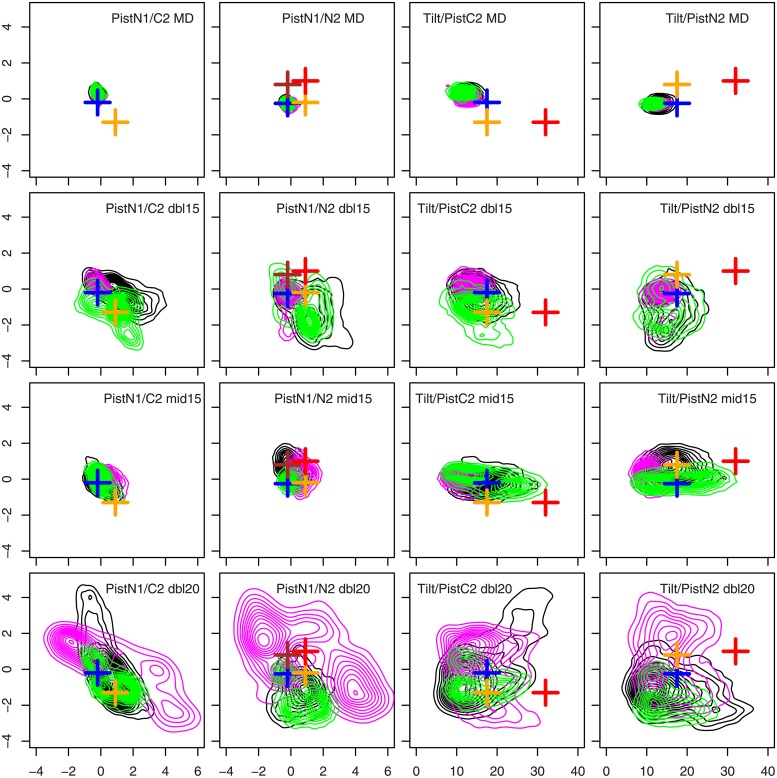
Relative motions of HAMP *α* helices. Contour plots describing the distribution of piston (Eq 5) and average tilt (Eq 7) along MD and TAMD trajectories dbl15, mid15 and tet20. Each plot is labeled XYT, where X and Y correspond to the x and y axis labels and T to the trajectory name. N1 and C2 refers to the names of HAMP helices given in Fig 1. The positions of the parameters for the representative conformations proposed by Zhu et al [41] on the isolated HAMP are displayed as crosses: P00 (blue), P01 (brown), P10 (orange) and P11 (red). On the plots Piston N1/C2, P00/P01 and P10/P11 are superimposed and only P00 and P10 are plotted. On the plots Average Tilt/Piston C2 and Average Tilt/Piston N2, P00/P01 are superimposed and only P00, P10 and P11 are plotted.

The parameters *Δ*_*pis*_ (Eq 5), and *θ*_*tilt*_ (Eq 7), introduced by Zhu et al [41] and described in Methods, were monitored along the trajectories. The joint distributions of parameters Piston N1/Piston C2, Piston N1/Piston N2, Average tilt/Piston C2, Average tilt/Piston N2, are plotted in Fig 3. In [41], these parameters vary in the following ranges: Piston N1 in -2/2 Å, Piston N2 in -2/2 Å, Piston C2 in -2/1.5 Å, Rotation N1 in -20/30°, Rotation C2 in -30/30°, Average Tilt in 0-35° (Fig 3 in [41]). In the present work, similar ranges are observed for MD and TAMD trajectories with artificial temperature of 15 kcal/mol (see Fig 3 for Pistons N1, N2, C2 and Average tilt). For Rotations N1 and C2, the range of variations are about -40/40° which is also similar to the observations in [41]. In trajectories tet20, the larger artificial temperature induces distribution wider than the ones observed in [41], meaning that a larger conformational space is sampled for HAMP in these trajectories.

This agreement for the *α* helices motions was put in parallel with the comparison of the primary sequences of the two HAMP domains. In the sequence alignment of Af1503-HAMP [17] and CpxA-HAMP obtained using TCoffee [63] (S5 Fig), 11 residues over 29 located in *α* helices, are conserved between the CpxA and the Af1503 sequences of HAMP. In addition, 18 residues over 43 are similar or very similar. The two HAMP sequences are thus quite similar, but far from being identical. One should notice that, in the present work, the collective variables were generic geometric centers of the Carbons *α* and have no specific relationship with the piston, rotation and scissoring parameters. In the work of Zhu et al [41], the collective variables were specific piston shifts, and rotation/tilt angles.

During the metadynamics analysis of HAMP conformational space [41], conformations P00, P01, P10 and P11 were extracted as representative conformations of isolated HAMP dimer. P00 represents the native state of HAMP in which no piston displacement has occurred; P01 and P10 represent states in which piston displacement has occurred respectively for the N2-C1 and N1-C2 pairs of HAMP *α* helices (Fig 1). In the state P11, both helical pairs have undergone a piston displacement. Using the piston displacements and average tilts given for the representative conformations in [41], the positions equivalent to P00, P01, P10 and P11 have been replaced in the distributions of Fig 3, and are drawn as colored crosses: P00 (blue), P01 (brown), P10 (orange) and P11 (red). The P00 cross (blue) is always sampled close to the center of distributions, whereas the other conformations are either superimposed on P00 (as P01) or only sampled along the TAMD trajectories. P01 (brown) is either superimposed on P00 and consequently not plotted (see caption of Fig 3) or only slightly aside from P00. The P10 (orange) is more apart from the middle of the distributions. All HAMP parameters of P11 (red) except the average tilt are sampled along TAMD trajectories. Interestingly, in the work of Zhu et al [41], the conformation P11 was shown to be metastable and to return to either P01 or P10 state in MD trajectories.

The parameters of the representative conformations closest to the distribution middle in the present work are those of P00 and P01, in which no piston displacement involves the N1-C2 helix pair [41]. This piston asymmetry reflects the asymmetry observed in the starting point 4BIV of simulations, in which the N1 and C2 *α* helices are blocked by the presence of the domain CA^−^ (Fig 1).

The software SamCC [34] was used to determine the gearbox state of HAMP. The profiles of Crick angle deviations averaged among trajectories (S6 Fig) are similar to the ones observed in the X-ray crystallographic structure [26], ie. a profile corresponding to the knobs-into-holes state. The stability of the gearbox state observed along the trajectories agrees with several observations in the literature, as the one made along the metadynamics exploration of the HAMP conformational space [41], or the one made on the recently determined X-ray crystallographic structure of histidine kinase [42]. Beside, one should notice that the structures displaying variations of gearbox state were initially obtained on an isolated HAMP domain [17, 32, 33] by modifying the protein primary sequence. Subsequent observations of various gearbox states have been observed [27, 28] on chimeric proteins.

The relative motion of HAMP *α* helices observed here are similar to those put in evidence in Ref. [41]. This similarity is interesting as the two HAMP sequences are not identical. Beside, no gearbox transition is observed for HAMP during all trajectories.

### HAMP motions are correlated to functional motions of the histidine kinase

The presence of DHp and CA domains in the present simulations allows to monitor histidine kinase motions related to the protein function and to connect them to the variations of *α* helix positions in HAMP. The relative positions of HAMP, DHp and CA domains were proposed [44] to undergo segmental motions in order to alternatively sample autokinase and phosphotransferase configurations for the two CA domains (S1 Fig). In the present work, these variations of positions are described from the ratio *θ*_*HAD*_/*θ*_HA*D_, of the angles HAMP-CA^−^-DHp and HAMP-CA*-DHp, which is equal to 1.1 in 4BIV. As it was pointed out in the section “Variability of the global architecture of CpxA”, a ratio larger than 1 corresponds to an asymmetric arrangement of HAMP and DHp positions whereas a ratio closer to 1 corresponds to a more symmetric HAMP/DHp architecture (S2 and S4 Figs).

In the following, the joint distributions of HAMP parameters and of the angle ratio *θ*_*HAD*_/*θ*_HA*D_ will be analyzed by focusing on the local maxima of these distributions in order to put in evidence the main tendencies in the variations of distributions.

**Fig 4:**
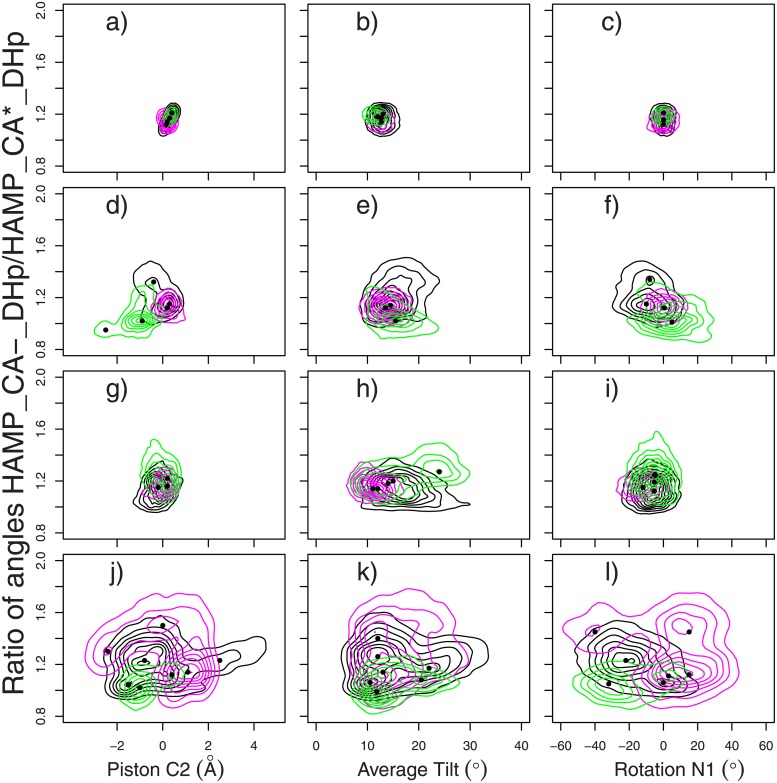
Correlation of HAMP motions to the ratio of the angles HAMP-CA^−^-DHp and HAMP-CA*-DHp. Ratio *θ*_*HAD*_/*θ*_HA*D_ of the angles HAMP-CA^−^-DHp and HAMP-CA*-DHp plotted along parameters describing the relative positions of HAMP *α* helices: Piston C2 (Eq 5), Rotation N1 (Eq 6) and average Tilt (Eq 7). The distributions are plotted for various trajectories: (a-c) MD, (d-f) dbl15, (g-i) mid15, (j-l) tet20. The contour plots of the distribution are colored according to the replicates, in the same way than in Fig 2. The locations of local maxima in distributions are marked with bullets.

Along MD trajectories, the ratio *θ*_*HAD*_/*θ*_HA*D_ is around 1.1 (Fig 4a–4c), the value observed in the starting point of trajectories: the X-ray crystallograpic structure 4BIV. During the TAMD trajectories, various correlation patterns are observed between the ratios and the different HAMP parameters. Considering the local maxima of distributions, largest ratio values (around 1.4) correspond to Piston C2 close to 0° for dbl15 (Fig 4d) and tet20 (Fig 4j). The local maxima located at increasing and decreasing values of C2 piston, display smaller ratio values down to 1. The Piston C2 displacement from the 4BIV conformation is thus correlated to smaller ratios and thus more symmetric HAMP/DHp architecture. Inverse trend is observed for Rotation N1. Indeed, the local maxima located at the zero value for Rotation N1 in dbl15 (Fig 4f) and tet20 (Fig 4l) display ratios in the 1.0-1.1 range, and for most of the positive and negative Rotation N1, larger ratios are observed. Moving the N1 rotation away from the position in 4BIV has thus a global tendency to increase the ratio, leading to the appearance of more asymmetric arrangements of HAMP and DHp within the dimer.

Average Tilt displays along dbl15 (Fig 4e) and tet20 (Fig 4h) a pattern different from the two other HAMP parameters. In dbl15 (Fig 4e), the local maxima are all located around tilt value of 15°, close to the one observed in MD, but correspond to various ratio values. In tet20 (Fig 4k), two tilt values, around 12 and 20°, are observed for various ratio values. In the presence of HAMP inter-monomer restraints dbl and tet (Table 3) connecting top and bottom HAMP residues, the average tilt seems thus to have a weaker correlation with the ratio than the other HAMP parameters.

**Table 3:** Characteristics of the distance restraints used along the TAMD trajectories to prevent the dissociation of HAMP monomers. The involved atoms are C*α* atoms of the residues given in the two first columns. The ranges of percentages of violations of these restraints along TAMD trajectories are given in the two last column: they correspond to the percentages of frames in which the distance is outside the restraint interval or outside of the interval increased by 10%.

Resid1	Resid2	force constant (kcal/mol)	lower bound (Å)	upper bound (Å)	percentage of violation	percentage violation 10%
dbl						
A-190	B-190	1.0	7.0	11.0	0.01-0.5	0-0.002
A-219	B-219					
A-204	B-204	1.0	13.0	17.0	0.02-0.25	0.0
A-230	B-230					
mid						
A-221	A-194	1.0	10.3	14.3	0.02-0.1	0-10^−4^
A 221	B-194	1.0	2.2	6.2	0.2-0.3	0.02-0.05
B-194	B-221	1.0	10.3	14.3	0.06,-0.1	0-10^−4^
A-194	B-221	1.0	2.2	6.2	0.1-0.2	0.01-0.03
tet						
A-190	A-219	1.0	10.0	14.0	0.2-0.5	0.001-0.002
B-190	B-219	1.0	10.0	14.0	0.1,0.4	0.001-0.01
A-190	B-219	1.0	7.0	11.0	0.2-0.3	0.01-0.02
A-219	B-190	1.0	7.0	11.0	0.1-0.3	0.002-0.01
A-204	A-230	1.0	9.0	13.0	0.1-0.4	0.0-0.005
B-204	B-230	1.0	9.0	13.0	0.3-0.4	0.001-0.003
A-204	B-230	1.0	13.0	17.0	0.2-0.3	0.0001-0.003
B-204	A-230	1.0	13.0	17.0	0.1-0.4	0.001-0.003

The distributions in the mid15 replicas (Fig 4g–4i), in which HAMP inter-monomer restraints mid (Table 3) connect residues located in the middle of HAMP, display trends inverse of those observed along other TAMD trajectories. Indeed, the local maxima for Piston C2 (Fig 4g) and Rotation N1 (Fig 4i) are quite concentrated around positions observed in MD (Fig 4a and 4c). At the contrary, for Average Tilt (Fig 4h), the positions of local maxima reveal a trend of increasing tilt along increasing ratio *θ*_*HAD*_/*θ*_HA*D_, which supports the hypothesis that increasing tilt would induce more asymmetry in the relative positions of HAMP and DHp.

Overall, the analysis of Fig 4 reveals correlation between relative motion of *α* helices in HAMP and relative positions of HAMP and DHp domains. In the segmental motion model [26, 44], the relative positions of HAMP and DHp are related to the functional transition of CpxA (S1 Fig). The HAMP *α* helices motions can be thus related to the CpxA function. Nevertheless, the presence of inter-monomer restraints within HAMP has an influence on the observation of motion correlations.

**Fig 5:**
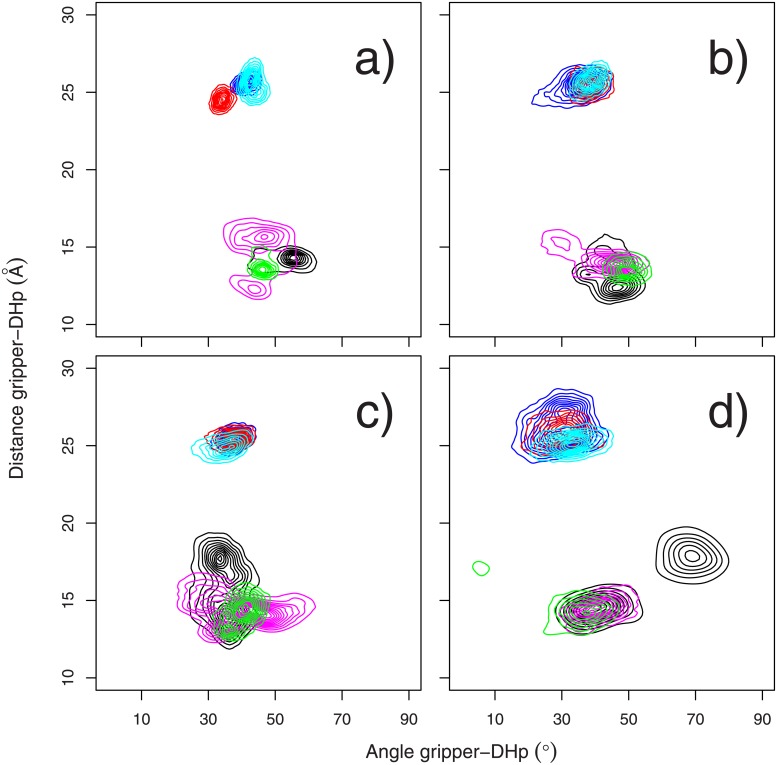
Analysis of the CpxA enzymatic state. C*α*-C*α* distances (Å) between N360 of region CA and H248 of region DHp plotted along the angles (°) between a gripper helix and its companion DHP helix ie: the gripper helix of CA* with helix 1A and the gripper helix of CA^−^ with helix 1B (Fig 1). The gripper helices are defined from residue G420 to Q430 in CA* and CA^−^ and the companion DHp *α* helices are defined from residue M235 to T255. Plots are given for the trajectories MD (a) and TAMD: dbl15 (b), mid15 (c) and tet20 (d), and on each plot the contours are colored according to the replicate. The distance between N360 of chain A (CA^−^) and H248 of chain B is plotted along the angle between the gripper helix of CA^−^ and the helix 1B of DHp using colors blue/red/cyan, whereas the distance between N360 of chain B (CA*) and H248 of chain A is plotted along the angle between the gripper helix of CA* and the helix 1A of DHp using colors black/magenta/green.

A recent review [64] proposed distances and angles to characterize the enzymatic state of histidine kinases basing on X-ray crystallographic structures. The distances are: the C*α*-C*α* distance between N360 from chain A and H248 from chain B and between N360 from chain B and H248 from chain A. The two angles are those between the axes of the gripper *α* helices (residues 420-430 in domains CA* or CA^−^) and the residues 235-255 in the *α* helices 1A or 1B in the DHp domain (Fig 1). The joint distributions of these distances and angles were plotted (Fig 5) in a way similar to those of the Fig 4 in Ref. [64] in order to analyse the enzymatic state.

Along all trajectories, the distances between N360(A)(CA^−^)-H248(B) and N360(B)(CA*)-H248(A) are respectively distributed around 25 and 13 Å (Fig 5a). The distance and angle values are close to the region where the Michaelis complex is observed in X-ray crystallographic structures analyzed by Bhate et al [64] (left plot of Fig 4 therein). Similarly, the DHp-gripper angles are around 40° which, according to [64], agrees with the auto-phosphorylation state of the domain CA* observed in the 4BIV structure [26]. The distributions of distances and angles get wider along TAMD trajectories (Fig 5b–5d), due to the larger internal mobility of the CpxA dimer. Interestingly, the distributions of angles and distances involving the catalytic domain CA* are all more dispersed (colors black/magenta/green) than the ones involving CA^−^ (colors blue/red/cyan). These CA* distributions display several peaks with DHp-gripper angle values in the range of 50-80° (Fig 5c and 5d), shifting toward the region of phosphatase-competent states in Fig 4b of Ref. [64], or with DHp-gripper distance values close to 20 Å (Fig 5c), shifting toward the region of inactive-like states (Fig 4b of Ref. [64]).

Within CA domains, the ATP position was further analyzed by calculating along each trajectory the average distances between atoms O*δ*1-2 of D386 sidechain and the phosphate group of ATP, as well as the minimum distance value between the H*η* hydrogens of the R363 sidechain and the O*γ* of ATP (Table 4). The average values and standard deviations of these distances show that the corresponding hydrogen bonds are formed during large parts of the trajectories. Consequently, although the ions Mg^2+^ were positioned in the catalytic site by superimposing the CA domains of 4BIV to those of 4BIW, the system reorganized and is able to establish interactions connecting the ATP to the CA residues.

**Table 4:** Average distances (Å) between D386 sidechain (O*δ*1-2) and the phosphate group of ATP, as well as the minimum distance value between the H*η* hydrogens of the R363 sidechain and the O*γ* of ATP, calculated along the replicated MD and TAMD trajectories. In the second line, the dissociation intervals are given along with the final distance value. The dissociation was supposed to take place for distances larger than 3 Å. If no dissociation occurs, a dash sign is written. **(d)** Equilibrium between a partially dissociated and bounded positions of ATP. **(f)** Few isolated dissociation events take place along the trajectory.

H-bond	MD	dbl15	mid15	tet20
CA^−^ ATP/D386	3.2 ± 0.2 -; 3.1 Å	3.3 ± 0.2 -; 3.0 Å	3.4 ± 0.2 -; 3.0 Å	5.1 ± 1.5 35-100ns; 6.7 Å
	7.4 ± 0.7 1-100ns; 7.1 Å	4.0 ± 1.0 80-100ns; 5.6 Å	3.3 ± 0.2 -; 3.1 Å	5.1 ± 1.1 29-100ns; 6.0 Å
	7.0 ± 3.1 5-30,59-100ns; 7.7 Å	3.3 ± 0.2 -; 3.2 Å	3.3 ± 0.2 -; 2.8 Å	3.3 ± 0.2 -; 3.4 Å
CA^−^ ATP/R363	1.7 ± 0.2 -; 1.6 Å	1.9 ± 0.4 20-50ns; 1.6 Å	1.7 ± 0.2 -; 2.0 Å	2.1 ± 0.8 65-80,95-100ns; 4.0 Å
	1.7 ± 0.1 **(f)**; 1.7 Å	1.8 ± 0.3 **(f)**; 1.9 Å	1.7 ± 0.2 -; 1.7 Å	1.8 ± 0.4 25-35ns; 1.6 Å
	2.2 ± 1.0 80-100ns; 4.3 Å	1.7 ± 0.2 -; 1.7 Å	1.9 ± 35-40ns; 1.8 Å	1.9 ± 0.6 10-20ns; 2.1 Å
CA* ATP/D386	3.4 ± 0.2 -; 3.3 Å	3.4 ± 0.2 -; 3.3 Å	6.2 ± 5.5 70-100ns; 26.4 Å	4.4 ± 0.8 **(d)**; 5.0 Å
	4.9 ± 0.9 18-100ns; 5.7 Å	3.3 ± 0.2 -; 3.3 Å	3.8 ± 0.9 80-100ns; 5.7 Å	3.4 ± 0.2 -; 3.4 Å
	3.4 ± 0.4 -; 3.3 Å	3.5 ± 0.2 -; 3.3 Å	3.4 ± 0.2 -; 3.6 Å	3.4 ± 0.2 -; 3.2 Å
CA* ATP/R363	1.7 ± 0.1 -; 1.7 Å	1.7 ± 0.1 90-100ns; 2.5 Å	1.9 ± 0.6 90-95ns; 1.8 Å	1.7 ± 0.2 -: 1.6 Å
	1.7 ± 0.2 **(f)**; 1.8 Å	1.7 ± 0.1 -; 1.6 Å	1.8 ± 0.2 -; 1.7 Å	1.8 ± 0.2 -; 1.6 Å
	1.8 ± 0.3 90-100ns; 2.5 Å	1.7 ± 0.1 -; 1.7 Å	1.7 ± 0.2 -; 1.7 Å	2.2 ± 1.1 90-100ns; 5.1 Å

In Table 4, it is also visible that in many cases, dissociation events are observed between the ATP and the residues D386 and R363. The times at which dissociations are observed are given along with the distance value in the last trajectory frame. These observations reveal that two replicas display simultaneous breaking of ATP/D386 and ATP/R363 interactions in CA^−^: the last replica of MD trajectory and the first replica of trajectory tet20, in the domain CA^−^. The domain CA^−^ being away from H248, it is not surprising that the interactions between ATP and CA residues are weaker.

For several replicas, only one of the two interactions ATP/R363 and ATP/D386 is broken. In CA^−^, the ATP/D386 interaction is broken twice in the second replica of dbl15 and tet20, whereas in CA*, it is broken four times: in the second replica of MD, in the first and second replicas of mid15, in the first replica of tet20. The ATP/R363 interaction is broken three times only in CA* for the last replica of MD and tet20, and for the first replica of dbl15. The ATP/D386 interaction seems thus more easy to break than the ATP/R363.

**Fig 6:**
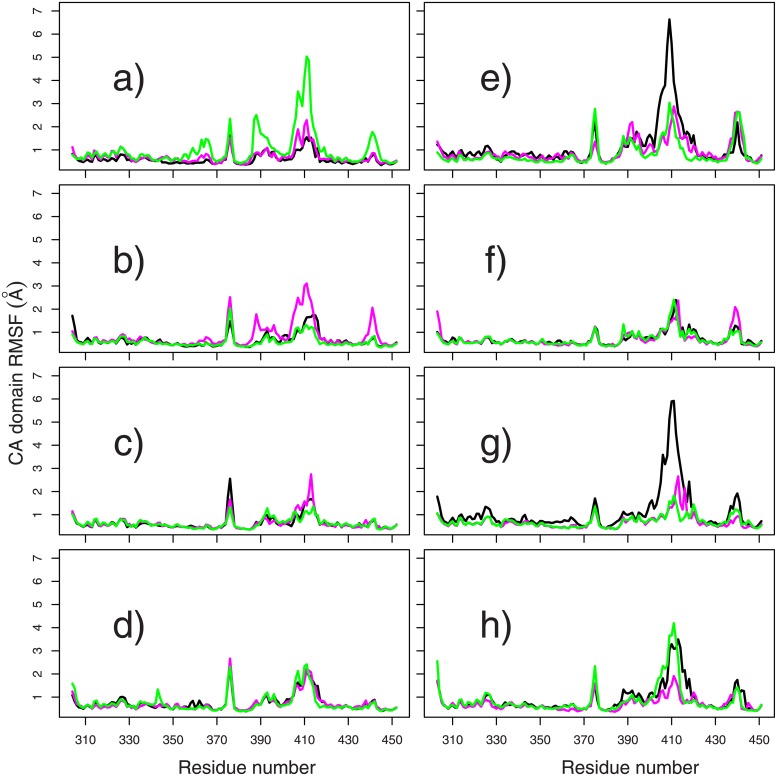
Atomic fluctuations by residues along the catalytic domains. Atomic fluctuations (RMSF: Å) plotted along the CA residues for the domains CA^−^ (a-d) and CA* (e-h). The calculations were performed on the replicas of trajectories: MD (a,d), dbl15 (b,e), mid15 (c,f) and tet20 (d,h), and the curves are drawn in the same colors than in Fig 2.

Internal mobility in the catalytic domains has been investigated by calculating the root-mean-square fluctuations (RMSF: Å) by residues for the domains CA^−^ and CA*, along each trajectory (Fig 6). Each CA domain was initially superimposed on the corresponding CA domain in the 4BIV conformation before calculating the fluctuations. Peaks of fluctuations and large variations of these fluctuations are located in the same regions, around residues 376-377, residues 387-397 (loop containing D386), residues 407-417 (loop containing the G2 box and the ATP lid) and residues 439-447. The G2 box and the ATP lid as well as the loop containing D386 form a part of the binding pocket of ATP, and their fluctuations are directly related to the variations of ATP/CA interaction previously described.

In CA^−^ (Fig 6a–6d), the atomic fluctuations do not vary much along the trajectories, except for the green replica of MD (Fig 6a), and the magenta replica of dbl15 (Fig 6b). These two peaks of fluctuations can be put in relation with the breaking of ATP/D386 interaction in the green replica of MD, and in the magenta replica of dbl15 (Table 4). In CA* (Fig 6e–6h), larger increase of fluctuations are observed than in CA^−^ mainly in the G2 box and the ATP lid, for MD (Fig 6e), mid15 (Fig 6g) and tet20 (Fig 6h). These variations are encountered in the first replica of MD, mid15 and tet20, and in the last replica of tet20, and correspond to the breaking of interaction ATP/D386 observed above in Table 4.

The relative motions of HAMP *α* helices have been correlated (Fig 4) to the variation of HAMP and DHp relative positions, described by the segmental motion model [26, 44]. Te analysis of the enzymatic state from distances and angles between CA and DHp domains (Fig 5) agrees with the initial states present in 4BIV. The analysis of CA domain positions reveals that CA* is more mobile than CA^−^. This is visible: (i) from the N360/H248 distance and the angles between gripper and DHp helices (Fig 5); (ii) from the internal fluctuations of the CA domains (Fig 2). This larger mobility of CA* agrees with the observations made from the superposition of X-ray crystallographic structures of CpxA [26].

## Discussion

The enhanced sampling approach TAMD has been used here to explore the conformational space of the histidine kinase CpxA. Collective variables have been chosen as geometric centers of Carbons *α* located in various protein regions: HAMP and DHp *α* helices and CA domains. Although the geometric centers of HAMP *α* helices are generic collective variables not related to specific relative motions of the helices, the use of such collective variables permitted to observe relative motions of the helices, similar to the motions observed along a metadynamics analysis of an isolated HAMP domain [41]. This point is interesting: indeed, the HAMP domain studied here was constrained by the presence of the other domains of histidine kinase, and could thus have a completely different behavior. This observation thus supports the hypothesis that HAMP has an intrinsic conformational behavior and can play the role of signal transmitter to influence the conformations of neighbouring domains.

Different motions of pistoning, scissoring and tilting for *α* helices within HAMP were observed, in agreement with the models proposed in the literature [17, 31, 64]. At contrary, no transition was observed between the two states of the gearbox model, as, along all trajectories, the HAMP conformation remains close to the knobs-into-holes initial state. A similar trend was observed for the metadynamics study of isolated HAMP [41] as well as for the structures of NarQ [42].

As in the present study the HAMP motions were simulated within the cytoplasmic domain of histidine kinase CpxA, the influence of these motions on the other CpxA regions could be put in evidence. Variations of pistons, rotations and tilt [41] were related to the relative orientations of HAMP and DHp domains [26, 44]. The geometric centers located on DHp and HAMP *α* helices also permitted to sample the relative orientations of HAMP and DHp domains. This has put in evidence global conformational tendencies of CpxA agreeing with the segmental motion of HAMP and DHp underlying the CpxA function [44] (S1 Fig). Both tendencies agree with the model proposed by [44], in which the CpxA CA domains alternate between different enzymatic states, each enzymatic state being populated in the presence of opposite orientations of HAMP and DHp.

The correlation of HAMP motions on the HAMP/DHp relative orientations is influenced by the type of restraints applied between HAMP residues. If restraints connect top and bottom HAMP residues, the movements of Piston C2 and Rotation N1 display opposite influences on the HAMP/DHp arrangement. On the other hand, if restraints connect middle HAMP residues, a effect of tilt motion is observed in agreement with the observations made on the X-ray crystallographic structure of NarQ [42].

The monitoring of the gripper helix orientation with respect to DHp has permitted to put in evidence tendencies of the gripper helix CA* to move away from the autokinase position. This tendency agrees with the motions of HAMP and DHp toward more symmetric positions.

To conclude, the present study permitted to confirm the existence of rotation, piston and scissoring motions of *α* helices in HAMP within the CpxA dimer. Furthermore, these motions were related to the more global segmental motion of HAMP and DHp [26, 44], connected to the functional states of this histidine kinase. The connection of HAMP motions to CpxA global motions related to the protein function represents an important step toward the understanding at the atomic level, of the signal propagation within the domains of histidine kinases.

## Methods

### System preparation

The homodimer of CpxA bound to ATP ligands has been taken from the X-ray crystallographic structure 4BIV (Fig 1) [26]. The ions Mg^2+^ were positioned by superimposition to the CA domains of 4BIW and the seleno-Methionines were replaced by Methionines. The CHARMM force field C36 [65–67] was used and 14 Na^+^ counter-ions were added to neutralize the system. The system was then hydrated in a box of 21233 TIP3P [68] water molecules in order to obtain a layer of water of 12 Å from any protein atom and in each direction. The total number of atoms was 72261.

Several collective variables (CVs) have been chosen to probe the relative internal dynamics of the different parts of the CpxA dimer (Fig 1). In HAMP and DHp, geometric centers of C*α* coordinates have been chosen as CVs in order to let the *α* helices free to undergo any relative motions. A first set of 4 CVs (H) is defined as the geometric centers of the four *α* helices forming the HAMP structure, ie: residues 188-201 and 219-234. A second set of 4 CVs (D) is defined as the geometric centers of the four *α* helices located in the DHp domain, ie: residues 235-266 and 272-299. The third set (C) are defined as the geometric centers of the CA* and CA^−^ catalytic domains, ie: residues 306-452. The overal set of these geometric centers, called HDC, was used as CVs in all TAMD trajectories.

### MD and TAMD trajectories

The temperature-accelerated molecular dynamics (TAMD) approach is an enhanced sampling approach, based on the parallel evolution of the protein coordinates ***x*** in a classical MD simulation (Eq 1) and of the target values ***z*** for the collective variables (CV) *θ*_*α*_(***x***) (Eq 2):

$$\begin{array}{c} M\ddot{x}=-\gamma \dot{x}-\nabla_{x}V\left(x\right)-\kappa \sum_{\alpha =1}^{N}\left(\theta_{\alpha }\left(x\right)-z_{\alpha }\right)\nabla_{x}\theta_{\alpha }\left(x\right)\\ \;+\sqrt{2M\gamma \beta^{-1}}\;\eta^{x}\left(t\right) \end{array}$$ (1)

$$\begin{array}{c} \bar{\gamma }\dot{z}=\kappa \left(\theta \left(x\right)-z\right)+\sqrt{2\bar{\gamma }{\bar{\beta }}^{-1}}\;\eta^{z}\left(t\right) \end{array}$$ (2)

where ***x*** are the physical variables (atomic coordinates) of the system, *θ*(***x***) are the current values of the collective variables and ***z*** the ever evolving target values of the collective variables. *M* is the mass matrix, *V*(***x***) is the empirical classical potential of the system, ***η***^*x*,*z*^(*t*) are white noises (i.e. Gaussian processes with mean 0 and covariance $<\eta_{\alpha }^{p}\left(t\right)\eta_{\alpha^{\prime }}^{p}\left(t^{\prime }\right)>=\delta_{\alpha \alpha^{\prime }}\delta \left(t-t^{\prime }\right)$, with *p* = ***x***, ***z***), *κ* > 0 is the so-called spring force constant, $\gamma ,\bar{\gamma }>0$ are friction coefficients of the Langevin thermostats, *β*^−1^ = *k*_*B*_*T*, ${\bar{\beta }}^{-1}=k_{B}\bar{T}$ with *k*_*B*_ the Boltzmann constant and $T,\bar{T}$ the temperatures.

Eqs 1 and 2 describe the motion of ***x*** and ***z*** under the extended potential

$$\begin{array}{c} U_{\kappa }\left(x,z\right)=V\left(x\right)+\frac{1}{2}\kappa {∥\theta (x)-z∥}^{2}. \end{array}$$ (3)

It was shown in [45] that by adjusting the parameter *κ* so that ***z***(*t*) ≈ ***θ***(***x***(*t*)) and the friction coefficient $\bar{\gamma }$ so that the ***z*** move slower than ***x***, one can generate a trajectory ***z***(*t*) in *z*-space which effectively moves at the artificial temperature $\bar{T}$ on the free energy hyper-surface *F*(***z***) defined at the physical temperature *T*. Then, using $\bar{T}>T$ in Eq 2 accelerates the exploration of the free energy landscape by the ***z***(*t*) trajectory, as energy barriers can be crossed more easily.

The trajectories were recorded using NAMD 2.7b2 [69]. The simulations were performed in the NPT ensemble. The friction coefficient, *γ* = 0.5 ps^−1^, and the physical thermal energy, *β*^-1^ = 0.6 kcal/mol, are the parameters of the conventional Langevin thermostat [70], allowing to obtain a simulation temperature of 300 K. The restraint force constant is set to *κ* = 100 kcal/(mol.Å^2^). The pressure was regulated with the Langevin piston Nose-Hoover method [71, 72], applying a pressure of 1 atm.

A cutoff of 12 Å and a switching distance of 10 Å were defined for non-bonded interactions, while long-range electrostatic interactions were calculated with the Particle Mesh Ewald (PME) protocol [73]. The SHAKE algorithm [74, 75] was used to keep rigid all covalent bonds involving hydrogens, enabling a time step of 2 fs. Atomic coordinates were saved every 10 picosecond. At the beginning of each trajectory, the system was first minimized for 1,000 steps, then heated up gradually from 0 K to 300 K in 30,000 integration steps. Finally, the system was equilibrated for 50,000 steps.

The 10 ns frame of one MD trajectory was the starting point of the TAMD enhanced sampling runs using the HDC collective variables. The TAMD approach was implemented in NAMD using a tcl script. Artificial temperature ${\bar{\beta }}^{-1}$ values of 15 and 20 kcal/mol have been employed. The artificial friction $\bar{\gamma }$ has been calibrated to 0.02ps^−1^, using the procedure described in [76], in which the running average *G*_*j*_(*N*) (S7 Fig) of the restraining force for each collective variable is computed along a 100 ps MD simulation in which the collective variables are restrained at their initial values:

$$\begin{array}{c} G_{j}\left(N\right)=\frac{\kappa }{N}\sum_{i=1}^{N}\left[\theta_{j}\left(x\left(t_{i}\right)\right)-z_{j}\right] \end{array}$$ (4)

where *θ*_*j*_(*x*(*t*_*i*_)) is the instantaneous value at the time *t*_*i*_ of the collective variable, *z*_*j*_ is the target value of the analyzed collective variable *j*, *κ* is the force constant.

The enhanced sampling trajectories were recorded in the presence of light restraints, applied using the colvars module of NAMD [77] and intending to prevent the dissociation of the HAMP dimer. These restraints are harmonic, applied on various distances between HAMP residues (Table 3). One should notice that they are violated in less than 0.5% of any trajectory duration and that most of the violations are within interval ranges increased by 10% (Table 3). Three types of restraints were applied: (i) dbl: two ambiguous distances between chains A and B, connecting the residue pairs 190/219 at the top of HAMP (close to the membrane side) and the residue pairs 204/230 at the bottom of HAMP (close to DHp); (ii) mid: four unambiguous restraints connecting residues A-221, A-194, B-221, B-194 located at the middle of HAMP domain; (iii) tet: eight non ambiguous restraints connecting, on one side the residues A-190, A-219, B-190, B-219, and on the other side the residues A-204, A-230, B-204, B-230. The restraints (i) and (ii) have been used with an artificial temperature ${\bar{\beta }}^{-1}$ equal to 15 kcal/mol to record the trajectories dbl15 and mid15. The restraint (iii) has been used with an artificial temperature ${\bar{\beta }}^{-1}$ equal to 20 kcal/mol to record the trajectory tet20. For each set of restraints, three replicas of 100-ns trajectories were recorded, to obtain along with three 100-ns replicates of MD trajectory a cumulative duration of 1.2 *μ*s.

### Analysis of HAMP and CpxA architecture

The state of the gearbox model was determined using SamCC [34] calculations on every frame of the trajectories. Other analyses have been performed using cpptraj [78]. Correlation analyses have been plotted as contour plots describing the joint probability distribution of two chosen variables.

Following the procedure proposed by Zhu et al [41], analyses were also conducted to characterize the relative positions of *α* helices in HAMP by geometrical parameters: piston displacement Δ_*pis*_, helix rotation angle *θ*_*rot*_ and tilt angle *θ*_*tilt*_, describing the helix scissoring motion between chains A and B (S3 Fig). Before conducting these analyses, each HAMP conformation was superimposed to the HAMP conformation in 4BIV, the parameters values correspond thus to displacements with respect to 4BIV.

The piston displacement Δ_*pis*_ and the rotation angles *θ*_*rot*_ are defined on each *α* helix as [41]:

$$\begin{array}{c} \Delta_{pis}=\frac{(R_{c}-R_{c}^{0}).R}{\left|R\right|} \end{array}$$ (5)

$$\begin{array}{c} \theta_{rot}=sign\left(R_{rc}.\left(R_{0c}\wedge R\right)\right)arccos\left(\frac{\left(R_{rc}\wedge R\right).\left(R_{0c}\wedge R\right)}{|R_{rc}\wedge R||R_{0c}\wedge R|}\right) \end{array}$$ (6)

where *R* = *R*_*h*_−*R*_*t*_, and *R*_*rc*_ = *R*_*r*_−*R*_*c*_. *R*_*h*_ and *R*_*t*_ are respectively the vectors connecting the head and the tail of an *α* helix of the HAMP domain. The helices are defined as: residues 189-204 and 217-232 in chains A and B, and are labeled N1, C1, N2, C2 in the same way than in [41] (Fig 1). The head and tail are defined as the four consecutive C*α* atoms at the N and C terminal extremities of the considered helix. *R*_*r*_ is defined by the center of mass of a series of C*α* atoms of residues 283, 287, 291 and 295 for helices N1 and N2, and of residues 313, 317, 321 and 325 for helices C1 and C2. *R*_*c*_ = (*R*_*h*_ + *R*_*t*_)/2 is the geometric center of the vector *R*. The vectors $R_{c}^{0}$, $R_{h}^{0}$, $R_{t}^{0}$ and $R_{r}^{0}$ are the corresponding vectors measured at the initial positions in the analyzed trajectory, and $R_{0c}=R_{r}^{0}-R_{c}$. The vectors *R*_*rc*_ and *R*_0*c*_ are defined as: *R*_*rc*_ = *R*_*r*_−*R*_*c*_ and $R_{0c}=R_{r}^{0}-R_{c}$. In the following, the piston displacement and the rotation angles will be denoted as Piston HH or Rotation HH, HH being N1, C1, N2, C2, depending on the analyzed *α* helix.

The tilt *θ*_*tilt*_ angle between the chains A and B is defined as:

$$\begin{array}{c} \theta_{tilt}=arccos\left(\frac{R_{i}.R_{j}}{|R_{i}\left|\right|R_{j}|}\right) \end{array}$$ (7)

where $R_{i}=R_{h}^{i}-R_{t}^{i}$ and $R_{j}=R_{h}^{j}-R_{t}^{j}$. The vectors with indexes *i* and *j* are calculated from the average positions of helix tails and heads respectively in chain A and B. In the following, the tilt angle will be denoted as Average Tilt.

## Supporting information

S1 Fig Scheme displaying the different functional states of CpxA. Two main schematic conformations of the CpxA histidine kinase dimer, displaying alternative positions of the two CA domains: the labeled CA* domains are in position to perform the autokinase reaction by transfering a phosphate group to H248, the labeled CA^−^ domains are away from H248 to allow the phosphotransferase reaction, transferring the phosphate group to the response regulator. The color code of the chain is the same than in Fig 1, whith the HAMP dimer in blue (chain A) and cyan (chain B), the DHp dimer in green (chain A) and yellow (chain B) and the CA domains of chain A and B respectively in red and magenta. The response regulator domains are labeled ‘REC’. Along the transition, the same CA domain populates alternatively positions corresponding to autokinase and phosphotransferase reaction. The scheme was inspired by the Fig 3 of [44].
(EPS)

S2 Fig Variation of the angles *θ*_*HAD*_ and *θ*_HA*D_. Contour plots describing the variation of the angle *θ*_*HAD*_ (HAMP-CA^−^-DHp) with respect to the angle *θ*_*HAD*_ (HAMP-CA*-DHp). The contour lines describes the joint probability distribution of the two angles, calculated from the coordinates of the geometric centers of involved domains: the four HAMP *α* helices spanning residues 189-203 and 217-231, the four DHp *α* helices spanning residues 240-268 and 271-298, and the CA^−^ and CA* domains spanning residues 305-453. The calculations are done on trajectories: (a) MD, (b) dbl15, (c) mid15, (d) tet20, and the contours are colored according to the replica, in the same way than in Fig 2. The straight line x = y is traced on the plots with a dashed line.
(EPS)

S3 Fig Parameters used for describing the CpxA conformations. The relative positions of *α* helices in HAMP are characterized by geometrical parameters: piston displacement Δ_*pis*_, helix rotation angle *θ*_*rot*_ and tilt angle *θ*_*tilt*_, describing the helix scissoring motion between chains A and B. The organisation of the whole structure was monitored by the ratio between the angles *θ*_*HAD*_ and *θ*_HA*D_. The angle *θ*_*HAD*_ is the angle between the geometric centers of domains HAMP, CA and DHp, whereas the *θ*_*HAD*_ is the angle between the geometric centers of domains HAMP, CA* and DHp. The scheme was partly inspired by the Fig 8 of [41].
(EPS)

S4 Fig CpxA conformations observed for extreme values of the ratio *θ*_*HAD*_/*θ*_HA*D_. For each trajectory, two conformations displaying angle ratio value within 10% (5% for MD) of the maximum or minimum values, have been extracted and are displayed colored in the same way than the curves in the Fig 2.
(TIFF)

S5 Fig Alignment of primary sequences for Af1503-HAMP and CpxA-HAMP. Alignement of protein sequences of Af1503-HAMP (PDB entries: 2L7H and 2L7I) and CpxA-HAMP (PDB entry: 4BIV) obtained using TCoffee [63]. The conserved residues are overlaid with black color.
(EPS)

S6 Fig Profiles of average Crick deviations (°). Average Crick angle deviation along with their standard deviations, plotted along the HAMP residue layer. The profiles were calculated using SamCC [34] along the first replica of MD (a) and TAMD trajectories dbl15 (b), mid15 (c) and tet20 (d). Each curve corresponds to one HAMP *α* helix: N1 (black), N2 (magenta), C1 (blue), C2 (green) according to the labels of Fig 1.
(EPS)

S7 Figdetermination of $\bar{\gamma }$ value.Plots of running average *G*_*j*_(*N*) of the restraining force projected on x (black curve), y (blue curve) and z (green curve) axes. for each collective variable on the geometric centers of HAMP and DHp *α* helices as well as of the catalytic domains CA. These parameters are plotted along the time, on 100-ps MD trajectories recorded without evolving the target values of the collective variables. The collective variables HAMP1A and HAMP1B correspond to the geometric centers of C*α* Carbones of residues 188-201 in chains A and B. The collective variables HAMP2A and HAMP2B correspond to the geometric centers of C*α* Carbones of residues 219-234 in chains A and B. The collective variables DHp1A and DHp1B correspond to the geometric centers of C*α* Carbones of residues 235-266 in chains A and B. The collective variables DHp2A and DHp2B correspond to the geometric centers of C*α* Carbones of residues 272-299 in chains A and B. The collective variables CAA and CAB correspond to the geometric centers of C*α* Carbones of residues 306-452 in the catalytic domains CA^−^ and CA* in chains A and B.(EPS)
