# Supplementary figures and images for: Conformational sampling of CpxA: Connecting HAMP motions to the histidine kinase function
Source: PLoS One. 2018 Nov 29;13(11):e0207899. doi: 10.1371/journal.pone.0207899 (PMC6264157; doi:10.1371/journal.pone.0207899)

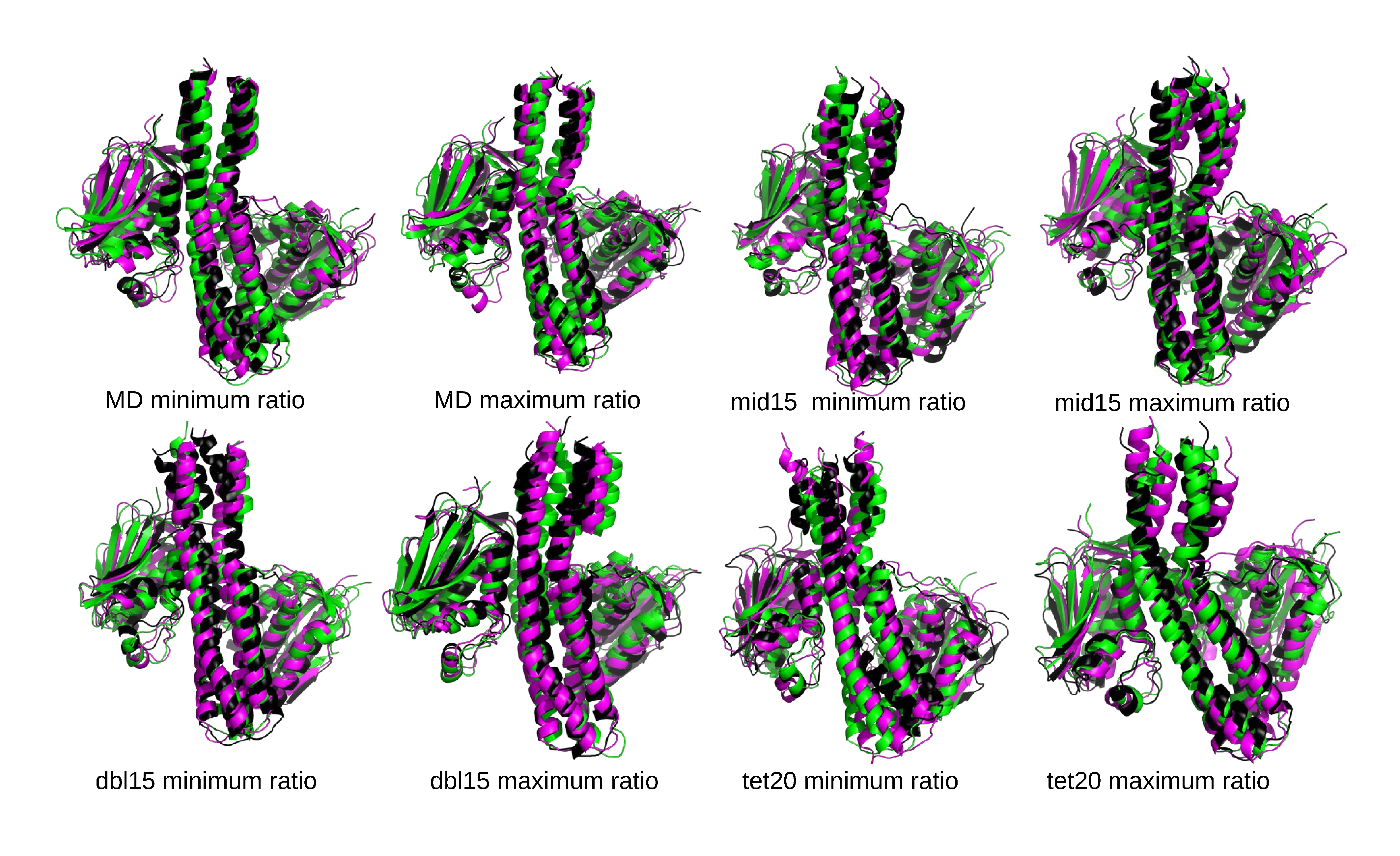

Supplement: S4 Fig — For each trajectory, two conformations displaying angle ratio value within 10% (5% for MD) of the maximum or minimum values, have been extracted and are displayed colored in the same way than the curves in the Fig 2. (TIFF) [file pone.0207899.s004.tiff]
